# Nurses' Perceptions and Experiences of Unethical Pro-Organizational Behavior: A Descriptive Qualitative Study

**DOI:** 10.1155/jonm/9948969

**Published:** 2025-07-23

**Authors:** Yue Li, Huan Chen, Xiaohui Dong, Jing Yang, Xinyu Chen, Di Gao, Dingxi Bai, Xianying Lu, Xuemei Xie, Chaoming Hou, Jing Gao

**Affiliations:** ^1^School of Nursing, Chengdu University of Traditional Chinese Medicine, Chengdu, Sichuan, China; ^2^Department of Geriatrics, Affiliated Hospital of Chengdu University of Traditional Chinese Medicine, Chengdu, Sichuan, China

**Keywords:** directed content analysis, nursing ethics, qualitative study, unethical pro-organizational behavior

## Abstract

**Background:** Unethical pro-organizational behavior (UPB) is detrimental to the organization's long-term growth and can even affect the health of the industry landscape. However, we know very little about the current UPB in nursing.

**Aim:** This study aimed to explore nurses' perceptions and experiences of UPB using the theory of planned behavior (TPB).

**Methods:** A descriptive qualitative study was employed. Fifteen nurses from four hospitals in Chengdu, China, were selected for semistructured interviews by purposive sampling from January 2024 to March 2024 to collect data. Directed content analysis was used for analysis.

**Results:** One hundred and two codes were developed and organized into six themes and 14 subthemes. The themes of this study are nurses' ambivalent attitudes toward UPB (beliefs about the benefits of UPB and beliefs about the harms of UPB), multiple social pressures on UPB decision-making (influence of organization, influence of leadership, and influence of coworkers), factors influencing UPB implementation (facilitating factors for UPB implementation and barrier factors for UPB implementation), complexity of UPB's behavior intentions (stick to principles and resist UPB, obedience to rules and authority, consideration of interests, and flexible decision-making), UPB in clinical contexts (concealment of negative information and violation of behavioral norms), and nurses' recommendations for managing UPB (strengthening system construction, providing multifaceted support).

**Conclusions:** While UPB does exist in nursing, it involves multiple influencing factors and motivations and can have complex consequences. To reduce the incidence of UPB, organizations should provide nurses with multifaceted support while strengthening UPB management.

## 1. Introduction

In recent years, unethical pro-organizational behavior (UPB) has become a prominent social issue [1]. UPB as actions are intended to promote the effective functioning of the organization or its members and violate core societal values, mores, laws, or standards of proper conduct [2]. Examples include deceiving customers to increase an organization's profits and withholding damaging information to maintain or enhance its reputation [3]. There are two core components to this concept [2]. First, UPB is unethical. The second is the intent behind the unethical behavior; UPB is concerned with behavior that benefits the organization, its members, or both.

UPB has three boundary conditions [2]: Firstly, UPB must be an intentional act, not an act done by mistake, error, or unconsciousness. Secondly, UPB is judged based on the starting point of the act rather than the outcome; as long as the starting point of the unethical act is for the sake of the organization or its members, it is UPB regardless of whether the outcome benefits the organization. Finally, unethical behaviors conducted primarily with the intention of benefiting the self alone, and not the organization or its members, would not be considered UPB. The current research on nurse behaviors has dealt chiefly with workplace bullying [4], toxic leadership behaviors [5], professional misconduct [6, 7], disruptive behavior [8], medical error reporting [9], counterproductive work behaviors [10], organizational citizenship behavior [11], and proactive work behavior [12]. The literature indicates that nurses' unethical behavior is typically self-interested, potentially harming the organization and its members, which fundamentally differs from UPB.

While UPB is subjectively suitable for the interests of employees and the organization, in actuality, it is detrimental to the long-term growth of the organization and can even affect the health of the industry landscape [2]. In addition, the harmful results of UPB include increased employee anxiety, guilt, incivility, and work-to-life conflict, reduced job satisfaction, individual performance, organizational citizenship behavior toward customers, and lower stock prices [13]. In the medical field, the existence of UPB not only reduces the level of social trust in medical institutions but also hinders the rational allocation of social medical resources and harms the public interest of society [14]. Therefore, UPB, as an urgent challenge, requires widespread attention and effective management measures to prevent and control it.

The antecedents of UPB involve three main factors, including individual, interpersonal, and organizational factors. Firstly, among individual factors, factors such as organizational identification, moral disengagement, and Machiavellianism can affect the emergence of UPB [15–17]. Secondly, among interpersonal factors, leadership style, colleagues' UPB, belongingness, and other factors can affect UPB [18–20]. Finally, among organizational factors, factors such as organizational climate, political environment, and performance pressure can also affect UPB [21–23]. While previous studies have provided valuable insights into the antecedents and consequences of UPB, they have been conducted primarily in a business context. Fewer studies have been conducted for the medical field, and relevant studies have only explored the factors influencing UPB from the quantitative research perspective, including the effects of a sense of organizational support [14], emotional labor [24], performance appraisal [25], and ethical leadership [26] on the UPB of nurses. However, quantitative research focuses on quantitative data analysis, making it difficult to delve deeper into the complexity behind subjective experiences and behaviors. In contrast, qualitative research focuses on an in-depth understanding of the internal characteristics, motivations, attitudes, and deeper reasons behind the behavior of the research subjects, which is essential for revealing the deeper issues of complex phenomena and individual behaviors. However, no researcher has used a qualitative approach to explore nurses' UPB. In summary, there is limited current knowledge about nurses' UPB. Therefore, to promote positive organizational development, uphold patient rights, and improve the quality of care, qualitative research is necessary to understand nurses' perceptions and experiences of UPB.

The theory of planned behavior (TPB) [27], one of the most well-known attitudebehavior relationship theories in social psychology, was proposed by Ajzen in 1985 and serves as a general model for predicting and explaining various types of behavior [28]. TPB hypothesizes that behavioral intention is the most direct influencing factor on behavior, which in turn is influenced by attitudes toward the behavior (favorable or unfavorable perception of the act and the expected result of taking the act), subject norm (influence of significant others or groups on individual behavioral decisions), and perceived behavioral control (individual perceptions of factors that facilitate or impede executive behavior) [27]. Given that TPB has good explanatory and predictive power for behavior [29], it is currently widely used in research on different behaviors and their mechanisms, including dietary behavior, medical screening behavior, learning behavior, and other behaviors [30–32]. In addition, TPB has also been used to analyze nurses' perceptions and experiences of various behaviors, including unsafe behaviors, delirium management behaviors, clinical alarm management behaviors, and other behaviors [33–35]. Therefore, TPB provides an effective explanatory framework for exploring nurses' UPB, helping to understand nurses' attitudes toward UPB, the factors influencing nurses' implementation of UPB, and nurses' UPB. Based on the content of this study, we adjusted the definitions of the five elements of TPB. Specifically, attitudes toward the behavior refer to nurses' beliefs and assessments of the pros and cons of adopting UPB. Subject norms reflect the influence of significant others or groups about whether nurses should take UPB, which may impact their decisions. Perceived behavior control refers to nurses' perceptions of factors that facilitate or impede the execution of UPB. Behavior intention refers to the judgment of the subjective probability of taking UPB by nurses, which reflects the nurses' willingness to act on taking UPB. Behavior refers to nurses' implementation of UPB in their clinical work. Under the guidance of TPB, this study can provide a theoretical basis for subsequent research and management of nurses' UPB, further enriching the scope of nursing ethics and UPB research.

## 2. Methods

### 2.1. Study Design

A descriptive qualitative study was adopted based on naturalistic paradigm. Since the descriptive qualitative study is the method of choice when straight descriptions of phenomena are desired [36], we used a descriptive qualitative study design. It provides a comprehensive and direct understanding of the nurse's UPB experience and perceptions. This study's report follows the Consolidated Criteria for Reporting Qualitative Research (COREQ) [37].

### 2.2. Participants

Using purposive sampling, nurses from four hospitals in Chengdu, China, were selected from January 2024 to March 2024 for interviews. Inclusion criteria were nurses who were licensed to practice nursing and were working in all types of medical institutions, while voluntary withdrawal was the only exclusion criterion. In order to gain a broad perspective, nurses with different years of experience, wards, positions, and education levels were selected for interviews.

### 2.3. Data Collection

Data were collected through semistructured interviews, either face-to-face or online. The face-to-face interviews were conducted in private rooms in the participant's ward, and the online interviews were conducted using WeChat's video call function. No one other than the participant or interviewer was present during the interview. All participants consented to and used Chinese audio recordings, which were made with a recording pen. The audio recordings were transcribed verbatim within 24 h of the interviews. To ensure consistency and accuracy of the study data, all interviews were conducted by the first author. She is a female graduate nursing student with systematic training in qualitative research methods and 4 years of clinical experience.

First, the researcher contacted participants who met the inclusion criteria by phone or in person, introduced the participants to the content and purpose of the study, and invited them to participate. Verbal informed consent was obtained from each participant before the start of the interview, and demographics were collected at the end. The interview guide was designed based on the TPB and was formulated and modified after group discussion and preinterviews with two participants. Five key issues were eventually included which are as follows: (a) attitude toward the behavior: What benefits and harms do you think UPB will bring? (b) Subject norm: What individuals or groups do you think to influence the occurrence of UPB? (c) Perceived behavior control: In your opinion, what facilitating or barrier factors exist for the occurrence of UPB? (d) Behavior intention: When faced with a situation where UPB may occur, how would you handle the situation? (e) Behavior: Please tell us about your experience of UPB or UPB you know exists in healthcare. Further clarification is provided through exploratory questions such as “Can you give me an example?” and “Can you explain more?”

In total, 15 interviews lasted anywhere from 20 to 83 min, with a median duration of 37 min. Data collection and analysis took place simultaneously, and after the 13th interview, no new information emerged, and data saturation was reached. Two additional interviews were conducted to ensure the data were saturated, and ultimately, no new codes emerged. The researcher took field notes during the interviews and did not conduct repeat interviews. Interviews were transcribed into text and returned to participants to confirm the accuracy of the content.

### 2.4. Data Analysis

This study used directed content analysis [38], and NVivo 14.0 software was used to code the text. Before analyzing the data, the researchers first identified key concepts based on TPB as the initial coding scheme, including attitude toward the behavior, subject norm, perceived behavior control, behavior intention, and behavior. However, it does not exclude the possibility of new themes emerging from the analysis process. The first author highlighted and coded semantic units relevant to the objectives of this study after reading the interview transcripts several times. Coding involves labeling the semantic units with a descriptive code close to the original text and on a low level of abstraction and interpretation [39]. This will decrease the risk of missing essential content. Codes are then assigned to predetermined or new themes based on similarities and differences. The coding team discusses and revises the code through weekly meetings until a consensus is reached. The coding team included a nurse with a bachelor's degree, four graduate nursing students, three doctoral nursing students, and two professors. All authors have experience in conducting ethical or qualitative research in nursing. The first author translated representative quotes, codes, subthemes, and themes into English.

### 2.5. Trustworthiness

In order to improve the trustworthiness of the study, the following strategies were used in this study [40]: We chose participants of different ages, wards, positions, and levels of education to increase the likelihood of elucidating the research question from all sides. To increase the credibility of this study, representative quotations from the transcribed text were presented in the findings. The findings also sought agreement among coinvestigators, experts, and participants. In addition, the study results were returned to the participants for inspection, and based on feedback from all participants, the results were consistent with the data they provided. Another aspect of trustworthiness is dependability. Dependability is addressed through open dialog within the research team. Trustworthiness also includes the question of transferability. To promote transferability, we give an unambiguous description of the context of the study, the selection and characterization of participants, and the data collection and analysis process. We have elaborated on the findings and added appropriate illustrations, improving transferability. In addition, the researcher continues to reflect on his or her position during the research process, thereby improving the objectivity and accuracy of the study.

### 2.6. Ethical Considerations

This study received ethical approval from the Ethics Committee of Jinniu District People's Hospital, Chengdu, China (Approval Number: QYYLL-2024-13). The study was conducted in strict adherence to the principles of the Declaration of Helsinki [41]. Nurses were invited to participate voluntarily, and all obtained verbal informed consent before the interview. Participants could refuse to answer any questions or withdraw from the study at any time. The anonymity of the participants was ensured by deidentifying the interview transcripts so that the nurse being interviewed is denoted by “N,” i.e., N1 refers to Nurse 1. We also encrypted the computers and associated data so that only study members could access the data.

## 3. Findings

### 3.1. Characteristics of the Participants

One of the nurses we contacted declined to participate in the study due to lack of time. The final participants in this study included 15 clinical nurses. They are between 26 and 43 years.  Table 1 shows the characteristics of the participants.

### 3.2. Themes, Subthemes, and Codes

During the data analysis process, 102 codes were developed and categorized into six themes and 14 subthemes. Among the six themes of this study, nurses' ambivalent attitudes toward UPB, multiple social pressures on UPB decision-making, factors influencing UPB implementation, complexity of UPB's behavior intentions, and UPB in clinical contexts are the main elements of TPB, while nurses' recommendations for managing UPB are the new theme generalized in this study.  Table 2 shows the themes and subthemes. All codes and their representative quotes can be found in Supporting Appendix A.

#### 3.2.1. Nurses' Ambivalent Attitudes Toward UPB

Most participants indicated that UPB would benefit organizations, colleagues, individuals, and patients, but it would also cause long-term damage to many parties. Two subthemes were identified: beliefs about the benefits of UPB and beliefs about the harms of UPB.

##### 3.2.1.1. Beliefs About the Benefits of UPB

On the organizational side, participants felt that adopting UPB was good for maintaining the organization's reputation, helped the organization to reduce disputes and compensation, and could help the organization to be profitable.

N8: “*The third one is the hospital reputation aspect because once you have an accident, all the other patients may not want to come to this hospital.*”

In terms of coworkers, participants reported that implementing UPB helped to safeguard coworker interests and helped to maintain interpersonal relationships with coworkers.

N14: “*The purpose of one is to defend a coworker from being held accountable.*”

In addition to benefiting the organization and other stakeholders, UPB can bring benefits to itself, including avoiding punishment, reducing psychological stress, reducing hassle, and creating a good image for itself.

N3: “*If I don't, there will be more trouble.*”

Participants also reported that UPB was beneficial in maintaining the nurse's role status and the nursepatient relationship. Moreover, nurses will pay more attention to the patient after implementing UPB to compensate for possible adverse effects. At the same time, the quality of care will be improved by nurses reflecting on their behavior or corrective measures taken at the hospital level. In addition, several participants indicated that they were motivated to take UPB by preserving the multiple interests of the organization, coworkers, themselves, and patients.

N2: “*For example, an open indwelling needle is not disposed of in time, and so on, to save on consumables; one is to save on the patient's expenses, and the other is to save on the department's expenses.*”

##### 3.2.1.2. Beliefs About the Harms of UPB

For the nurses themselves, the implementation of UPB interferes with their rest, causes psychological distress, and can produce a fluke mind. Some participants feared that if UPB was discovered, they would be criticized, have their performance and bonuses docked, have extra work added, or even be unable to continue working in the nursing profession or go to jail.

N3: “*Bad results include giving me disciplinary action, and maybe not being able to work in the industry for the rest of my life, or going to jail.*”

For patients, the implementation of UPB by nurses can violate patients' interests, influence their medical decisions, delay their treatment, and may lead to psychological problems.

N10: “*The worst thing that can happen is physical harm to the patient, right? Prolonging the patient's treatment cycle and increasing hospitalization costs are all possible.*”

Some participants indicated that if a coworker implemented UPB, it would decrease trust in that coworker and affect job satisfaction. For the organization, implementing UPB by nurses can lead to disorganized management, damage to the organization's reputation, and long-term damage to the organization.

N13: “*Suppose this kind of thing ends up being exposed. In that case, both the department and the hospital will be subjected to public pressure from the community, some criticism, accusations, and suspicions, which will affect the reputation of the department and the hospital.*”

#### 3.2.2. Multiple Social Pressures on UPB Decision-Making

In the behavioral decision-making process, nurses are influenced not only by organizational pressures but also by their leaders and colleagues. Three subthemes were identified as follows: influence of organization, influence of leadership, and influence of coworkers.

##### 3.2.2.1. Influence of Organization

Some participants indicated that the organization's rules about whether nurses can adopt UPB affect their behavioral decisions.

N1: “*For unethical pro-organizational behavior, since the organization has told you not to do that, I think it shouldn't be done.*”

##### 3.2.2.2. Influence of Leadership

The majority of participants indicated that leaders' perceptions of UPB, as well as leaders' requirements for nurses to be able to take UPB, were crucial factors influencing behavioral decisions.

N1: “*This kind of coworker will not significantly influence me; the main thing is that the leader gives the word; for me, if the leader gives the word, I will listen a little bit.*”

##### 3.2.2.3. Influence of Coworkers

Some participants perceived coworkers' UPB and the need to maintain good relationships with coworkers as influencing their behavioral decisions.

N9: “*Coworkers influence each other because everyone thinks he is doing it, so why shouldn't I? It is all the same.*”

#### 3.2.3. Factors Influencing UPB Implementation

Among the promoting factors, multiple factors such as interpersonal relationships, management style, and multistakeholder interest considerations are intertwined, which jointly affect the occurrence of UPB in nurses. However, factors such as institutional norms, moral cultivation, and occupational well-being will hinder the occurrence of UPB in nurses. Two subthemes were identified: facilitating factors for UPB implementation and barrier factors for UPB implementation.

##### 3.2.3.1. Facilitating Factors for UPB Implementation

Most of the participants indicated that if nurses have a strong sense of identification with the hospital and a sense of collective honor, they will promote themselves to adopt UPB to maintain the reputation and interests of the department and the hospital. Secondly, participants indicated that if they had a good relationship with their coworkers, nurses would also promote themselves to take UPB to safeguard their coworkers' interests.

N1: “*I think they kept this under wraps to protect the department's interests, or even the hospital as a whole.*”

At the same time, some participants indicated that they would also be motivated by self-interest, not wanting to be fined, and fear of losing their jobs. Some personality traits also promote UPB, including a strong face-saving outlook and a high future-focused population that believes the interests of nurses and hospitals are aligned.

N12: “*It is more relevant to everyone's interests; if your hospital develops well, your department can develop well. Then, people can earn more and have more stable jobs. Moreover, with the current employment environment, people worry more about unemployment.*”

In addition to profit considerations, nurses' lack of adequate responsibility, professional quality, and a low level of awareness about UPB are contributing factors to the behavior. Regarding departmental climate, participants reported that some leaders were underaware of UPB and tolerant of the behavior, and individual leaders would ask nurses to perform UPB. Coworkers also implement UPB, and there can be cover-ups between coworkers, leading to a lack of ethical climate within the organization. As a result, nurses are also influenced by the atmosphere of the entire department, which promotes UPB. Some participants also indicated that managers' overly strict management styles and the lack of practical implementation of management measures were contributing factors to UPB.

N13: “*I think one of the first things is the lack of responsibility on the part of the nurses, the lack of professionalism, the kind of laziness of the nurses, and then the lack of appreciation for the potential consequences of this kind of thing.*”

N6: “*You know that is often not right, but maybe the whole department is like that … In order to fit in and then maybe slowly assimilate.*”

Most participants believed that to cope with the performance pressure on the department, they would resort to UPB to achieve cost savings in the department and increase the performance of the nurses. In addition, the high clinical workload, the mismatch of healthcare resources, especially the lack of human resources, and the cumbersome process of dealing with adverse events can also contribute to the occurrence of UPB. On the patient side, some participants indicated that patients' lack of understanding of nursing care and tensions in the nursepatient relationship were also facilitating factors. Tensions between doctors and nurses are also a facilitating factor.

N9: “*No one cares that much when the workload is so heavy; there is no way around it. No one cares about that much when they are so busy.*”

##### 3.2.3.2. Barrier Factors for UPB Implementation

Most participants indicated that strict quality control and institutional norms in the department, well-established supervision, and mechanisms to encourage reporting were barrier factors to UPB.

N3: “*Unless the department expressly forbids it from being done, I don't do it.*”

Some participants indicated that good ward managers help nurses solve problems, which reduces the incidence of UPB. By sharing their experiences of adverse events, nurses can increase risk awareness among young nurses, which contributes to the reduction of UPB.

N5: “*The nurse manager will help us from top to bottom with her resources, and most of our problems are reported to the nurse manager … So, having an excellent ward manager is a great thing.*”

In addition, the ethics-related education nurses receive in school is also a barrier factor for UPB. When faced with ethical decision-making conflicts, nurses' adherence to their own professional ethics and bottom line will reduce the occurrence of UPB. On the emotional side, nurses' strong sense of professional well-being and lack of belonging to the organization were also barrier factors.

N5: “*Barrier factors are certainly basic professional ethics; after all, I have also read for so many years that you are a clinical instructor, and of course, you should give others an example.*”

#### 3.2.4. Complexity of UPB's Behavior Intentions

Most participants consider the impact of UPB on patients, themselves, and their interpersonal relationships when deciding whether to adopt UPB and make flexible decisions based on specific circumstances. Some participants stuck to their principles and opposed the adoption of UPB. Three subthemes were identified: stick to principles and resist UPB, obedience to rules and authority, and consideration of interests and flexible decision-making.

##### 3.2.4.1. Stick to Principles and Resist UPB

Some participants indicated that when faced with a situation where UPB might occur, they would act on their principles rather than take UPB. Also, they will stop other's UPB.

N12: “*A matter of principle must not be committed, and if a matter of principle is committed, he must be reminded of the need to rectify the situation and then come with him to make amends.*”

##### 3.2.4.2. Obedience to Rules and Authority

Some participants expressed adherence to departmental default rules to maintain interpersonal relationships as long as UPB within the organization does not seriously impact patients. In addition, some participants reported that when there is a problem, nurses usually report it to senior nurses or leaders, who judge the severity of the matter before deferring to them in deciding whether to take UPB.

N8: “*Anything I can't handle I will ask the team leader because the team leader is more experienced, and then the team leader will also make a judgment. If the team leader thinks the matter is okay and will not affect the patient, we may be prepared to solve the problem internally.*”

##### 3.2.4.3. Consideration of Interests and Flexible Decision-Making

Some participants indicated that they would not take UPB if it would be a risk to themselves if they took it. One participant stated that if he was in a predicament and UPB happened to be the solution, he would not rule out taking UPB.

N3: “*I would probably be less likely to take this approach if I predicted there would still be a risk if I handled it this way.*”

Most participants indicated that they would take the behavior on a case-by-case basis if UPB did not affect the patient. However, the behavior would not be taken if UPB had severe consequences for the patient. Some participants also indicated that they would not stop a coworker's UPB because they did not know what position to take to stop another person's UPB and maintain good interpersonal relationships.

N5: “*If the consequences are severe, this behavior will be prevented. Indeed, the safety and value of the patient's life come first.*”

#### 3.2.5. UPB in Clinical Contexts

In a medical setting, the most common form of UPB involves nurses concealing negative information and violating work conduct norms to protect the interests of the organization and other stakeholders. Two subthemes were identified: concealment of negative information and violation of behavioral norms.

##### 3.2.5.1. Concealment of Negative Information

Some participants indicated that in their clinical work, they would conceal medical errors to protect the organization's interests. In addition, participants indicated that from time to time, they would help their coworkers conceal less severe incidents of error.

N9: “*If there is an adverse event in the department, you mustn't say anything about it for the organization's reputation.*”

##### 3.2.5.2. Violation of Behavioral Norms

Some participants indicated that they would violate the operation specification to save the department's cost or avoid the department being penalized. There are also times when nurses will tamper with nursing paperwork to avoid the department being penalized.

N11: “*Someone told me that the needleless connector for infusion in their department costs money. However, they can't charge for it, so they use the heparin cap to insert it. Patients are all chemotherapy patients, and there are many chemotherapy drugs, so it's very easy for them to leak.*”

#### 3.2.6. Nurses' Recommendations for Managing UPB

Organizations should pay attention to and strengthen the management of UPB, standardize nurses' behavior by formulating systems, clear rewards and punishments, and increase moral construction. At the same time, organizations should provide nurses with multifaceted support, including problem-solving, ensuring adequate human resources, resolving consumables issues, and other means to promote correct behavioral decisions by nurses. Two subthemes were identified: strengthening system construction and providing multifaceted support.

##### 3.2.6.1. Strengthening System Construction

Some participants indicated that organizations should implement strict quality control and develop systems to constrain the occurrence of UPB. Organizations can also manage the behavior by encouraging the reporting of UPB and setting strict criteria for rewards and penalties. Participants also noted that nurses should pay attention to the institutional requirements of the hospital and implement them effectively in their clinical work.

N13: “*This is definitely something that encourages whistle-blowing. First, management should set strict reward and punishment standards. Let everyone see this kind of thing happen after the attitude, and then the following talent will not take the risk.*”

Participants felt that ethics should also be strengthened, and relevant training should be added to increase nurses' awareness of the risks of UPB.

N9: “*I think the first thing is to have this awareness in the minds of the teachers in the clinic. First, let us know that this is an unregulated unethical behavior.*”

##### 3.2.6.2. Providing Multifaceted Support

In addition to strengthening the management of UPB, organizations should provide more organizational support for nurses. Participants indicated that when they encountered problems, they wanted the organization to help provide solutions. Some participants also indicated that ensuring adequate human resources and addressing the issue of consumables could help nurses avoid performing UPB because of excessive workload or save costs for the department.

N11: “*The first one is to have enough human resources so that when you reach a certain bed care ratio, you can take your time to do these things. This will prevent you from having too many things to do, and you will not be able to do them well in all areas.*”

Leaders should avoid making too strict management measures or blindly criticizing nurses to help nurses reduce their psychological burden and make the right behavioral decisions. Leaders and senior nurses in the unit should set a good example for young nurses and help them develop the right concepts.

N12: “*On the one hand, for example, when a nurse first comes in … We other nurses and our administrators have to give him the right message about what is okay to do and what is not okay to do, which is equivalent to teaching by example.*”

## 4. Discussion

To our knowledge, this study is the first qualitative study of UPB in nurses. Most previous studies have focused on the antecedents of UPB and less on the consequences [13]. The results of this study will help increase knowledge of the consequences of UPB. In terms of UPB benefits, the results of this study indicate that UPB is beneficial in preserving the interests and reputation of the organization and colleagues, and it is beneficial in maintaining interpersonal relationships, consistent with previous studies' results [13]. However, some participants felt that the benefits of UPB might also be skewed toward self-interest, such as preventing themselves from being penalized and reducing hassle. Another study also confirmed the role played by self-interested motivation in motivating UPB [42]. In addition, after implementing UPB, nurses reported being more attentive to the patient's condition, facilitating the maintenance of the nursepatient relationship. Therefore, this study indicates that nurses may adopt UPB to safeguard the interests of multiple parties, including the organization, colleagues, themselves, and patients, which goes beyond the organization's and colleagues' traditional motivation [2]. Managers should comprehensively consider these diverse motivations when developing relevant systems and measures to reduce nurse UPB more effectively.

Regarding the harms of UPB, the results of this study showed that UPB causes psychological harm, violates patients' interests, and delays their treatment. In addition, UPB can also cause physical and psychological harm to nurses, as well as financial losses to nurses, such as those that may be associated with fines, revocation of professional licenses, and other legal consequences. On an organizational level, UPB can cause long-term damage to the organization, leading to confused management and damage to the organization's reputation. This is consistent with the results of previous studies of other misbehaviors [6, 43–45]. Previous research has shown that UPB can also lead to work-life conflict [46]. The results of this study suggest that although UPB has short-term benefits, the harm produced by UPB is severe and long-term. Therefore, nurses' awareness of the consequences of UPB should be strengthened in the future to help them properly weigh the pros and cons. Some studies also point out that emphasizing the long-term harms caused by UPB may be a way for managers to discourage UPB [13].

The current research on the antecedents of UPB has focused on exploring facilitating factors. Previous research suggests that organizational identity is the most extensively studied antecedent of UPB to date [13]. However, in this study, high clinical workload, performance pressures on the department, and lack of awareness about UPB among nurses were the most frequently mentioned facilitators of UPB by nurses rather than organizational identity. Some studies have also shown that high workload has been identified as the most important organizational factor associated with unsafe practices [47]. Another study confirmed a positive correlation between performance stress and UPB [23]. Previous studies have also shown that moral disengagement [48], obsessive passion for work [49], Machiavellianism [17], psychological entitlement [50], loneliness in the workplace [51], feelings of indebtedness to the organization [52], and organizational commitment [53] are also the facilitators of UPB.

The barrier factors to UPB in this study mainly included sound management methods, such as strict quality control, institutional norms, and so on, as well as good ward managers, which were consistent with previous studies' results [18]. However, some research suggests that the relationship between ethical leadership and UPB may be curvilinear [54]. And the impact of different leadership styles on UPB in business has been shown [55, 56]. However, the effect of different leadership styles in the healthcare field on nurses' UPB is yet to be explored. Secondly, in terms of nurses' education, ethical education, and training in UPB risk awareness are barrier factors for UPB. In addition, on the emotional side, a strong sense of occupational well-being and a lack of belonging to the organization are also impediment factors for UPB. Because of the many factors that influence UPB in nurses, managers should comprehensively consider the multifaceted factors mentioned in the results of this study when developing strategies to prevent UPB in the future.

While UPB may be viewed as unethical by some, a better understanding of the conditions under which UPB is considered ethical or acceptable can help researchers predict when UPB is likely to occur [13]. In this study, nurses' behavioral intentions toward UPB mainly depended on whether UPB would result in serious adverse outcomes for patients. Most nurses indicated UPB is acceptable if it has no consequences for the patient. Only a few nurses said they would stick to their principles and not take UPB. This study also summarizes the specific behaviors of nurses UPB. Nurses' UPB can be divided into two types: nurses withholding negative information from outsiders to safeguard the organization's interests and other stakeholders within the organization and violating the norms of conduct at work. Previous studies have shown that misconduct in healthcare can damage the reputation of the nursing profession and the organization [57, 58]. Therefore, managers should take nurse UPB seriously and identify and manage it.

The results of this study suggest that strengthening system construction, as well as the provision of multifaceted support for nurses, is considered effective measures for managing UPB. Organizations should implement rigorous quality control, develop relevant systems, establish criteria for rewards and penalties, and encourage whistleblowing. Previous studies have also noted that misconduct can be reduced by appropriately assigning supervisors to supervise at different times and establishing protocols for reporting misconduct [6, 7]. Some studies have indicated that an effective incentive and disciplinary system is one of the effective factors in improving the professional ethics of nurses [7]. However, nurses indicated that systems should be implemented effectively in addition to developing systems, otherwise, problems will persist. In addition, organizations and schools should strengthen the training of nurses in ethics. Therefore, in the future, ways to better incorporate UPB into nursing ethics education should also be explored to increase nurses' awareness of the risks of UPB.

To manage UPB, the organization should also provide support to nurses in many ways. When problems are encountered, organizations and managers should help nurses find solutions and avoid blindly criticizing nurses or taking overly strict management measures. These measures will help to reduce the burden on nurses' minds and thus help them to make correct ethical decisions. In addition, inadequate human resources prevent nurses from providing optimal care, often leading to ethical dilemmas [59]. Therefore, the organization should also ensure that human resources are adequate and address the issue of consumables so that nurses do not implement UPB due to high workload or to save costs in the department. Consistent with this finding is a study showing that organizations can prevent misconduct by providing, managing, and optimizing resources [7]. Leaders and senior nurses on the unit should also lead by example and set the right concepts for novice nurses. For example, sharing negative experiences in the organization improves professional behavior and patient safety [6]. Additionally, previous research has shown that fostering an ethical climate in an organization will help reduce the incidence of UPB [18].

## 5. Limitation

First, due to time and funding constraints, we also lack the perspective of nurses in long-term care facilities and community hospitals, who may have new insights. Secondly, the generalizability of the findings is limited as this study is qualitative. Considering the influence of different cultures, there may be some variation in UPB in different countries. Future research should, therefore, explore nurses' perceptions and experiences of UPB in different countries and regions to gain comprehensive insights.

## 6. Conclusions

This study reveals the specific behaviors of nurses' UPB and their multidimensional influences and complex consequences. The study noted that while UPB may provide some benefit in the short term, the long-term harms are severe, and therefore, nurses' awareness of the consequences needs to be increased. At the same time, comprehensive management measures were proposed, including strict quality control, institutional standardization, education and training, and the provision of multifaceted support. The results of this study may increase the understanding of UPB in nursing, provide a theoretical basis for subsequent research and management of related behaviors, and further enrich the scope of nursing ethics and UPB research.

## Figures and Tables

**Table 1:** Characteristics of the participants.

No.	Gender	Age (years)	Educational level	Work experience (years)	Ward	Profession
N1	Female	27	Master's	4	Oncology department	Nurse
N2	Female	28	Bachelor's	6	Emergency	Nurse
N3	Male	28	Bachelor's	6	Anesthesiology	Nurse
N4	Female	34	Bachelor's	13	Gynecology	Nurse
N5	Female	31	Master's	7	Gynecology	Nurse
N6	Female	28	Bachelor's	6	Orthopedics	Nurse
N7	Female	36	Bachelor's	15	Hemodialysis room	Head nurse
N8	Male	28	Bachelor's	6	Intensive care unit	Nurse
N9	Female	43	Bachelor's	23	Obstetrics	Nurse
N10	Female	32	Bachelor's	11	Geriatrics	Nurse
N11	Female	26	Master's	2	Hematology	Nurse
N12	Female	33	Bachelor's	11	Neonatal intensive care unit	Nurse
N13	Male	28	Bachelor's	6	Emergency	Nurse
N14	Female	26	Master's	4	Oncology department	Nurse
N15	Female	38	Bachelor's	17	Orthopedics	Head nurse

**Table 2:** The subthemes and themes of the study.

Themes	Subthemes
Nurses' ambivalent attitudes toward UPB	Beliefs about the benefits of UPB
	Beliefs about the harms of UPB

Multiple social pressures on UPB decision-making	Influence of organization
	Influence of leadership
	Influence of coworkers

Factors influencing UPB implementation	Facilitating factors for UPB implementation
	Barrier factors for UPB implementation

Complexity of UPB's behavior intentions	Stick to principles and resist UPB
	Obedience to rules and authority
	Consideration of interests and flexible decision-making

UPB in clinical contexts	Concealment of negative information
	Violation of behavioral norms

Nurses' recommendations for managing UPB	Strengthening system construction
	Providing multifaceted support

## Data Availability

The data that support the findings of this study are available in the supporting information of this article.

## Supporting Information

All codes and their representative quotes can be found in Supporting Appendix A.

## References

[1] Zhao M., Qu S. (2022). Research on the Consequences of Employees’ Unethical Pro-Organizational Behavior: The Moderating Role of Moral Identity. *Frontiers in Psychology*.

[2] Umphress E., Bingham J. (2011). When Employees Do Bad Things for Good Reasons: Examining Unethical Pro-Organizational Behaviors. *Organization Science*.

[3] Umphress E. E., Bingham J. B., Mitchell M. S. (2010). Unethical Behavior in the Name of the Company: The Moderating Effect of Organizational Identification and Positive Reciprocity Beliefs on Unethical Pro-Organizational Behavior. *Journal of Applied Psychology*.

[4] Skarbek A. J., Johnson S., Dawson C. M. (2015). A Phenomenological Study of Nurse Manager Interventions Related to Workplace Bullying. *The Journal of Nursing Administration*.

[5] Zhou Y., Lin J., Liu X., Gao S., Yang F., Xu H. (2024). Validity and Reliability of the Toxic Leadership Behaviors of Nurse Managers Scale Among Chinese Nurses. *Frontiers in Psychology*.

[6] Varaei S., Nayeri N. D., Sayadi L., Shahmari M., Ghobadi A. (2024). Outcomes of Professional Misconduct by Nurses: A Qualitative Study. *BMC Nursing*.

[7] Ghobadi A., Sayadi L., Nayeri N. D., Shabestari A. N., Varaei S. (2024). The Nurses’ Perception of the Factors Influencing Professional Misconduct: A Qualitative Study. *Nursing Ethics*.

[8] Moreno-Leal P., Leal-Costa C., Díaz-Agea J. L. (2021). Disruptive Behavior at Hospitals and Factors Associated to Safer Care: A Systematic Review. *Healthcare (Basel, Switzerland)*.

[9] Namadi F., Alilu L., Habibzadeh H. (2024). Nurses’ Experiences of Reporting the Medical Errors of Their Colleagues: A Qualitative Study. *BMC Nursing*.

[10] Elliethey N. S., Aly Abou Hashish E., Ahmed Mohamed Elbassal N. (2024). Work Ethics and Its Relationship With Workplace Ostracism and Counterproductive Work Behaviours Among Nurses: A Structural Equation Model. *BMC Nursing*.

[11] Chu C. I., Lee M. S., Hsu H. M., Chen I. C. (2005). Clarification of the Antecedents of Hospital Nurse Organizational Citizenship Behavior: An Example From a Taiwan Regional Hospital. *Journal of Nursing Research*.

[12] Pierre L., Cangialosi N., Déprez G. R. M. (2024). Nurse Middle Managers’ Proactive Work Behavior: Antecedents and Consequences on Innovative Work Behavior and Job Performance. *Journal of Health Organization and Management*.

[13] Mo S., Lupoli M., Newman A., Umphress E. (2022). Good Intentions, Bad Behavior: A Review and Synthesis of the Literature on Unethical Prosocial Behavior (UPB) at Work. *Journal of Organizational Behavior*.

[14] Lu Z. J. (2022). *Research on the Influence of Perceived Organizational Support on Unethical Pro-Organizational Behavior of Medical Staff-Taking Organizational Identification as the Intermediary*.

[15] Alniacik E., Erbas Kelebek E. F., Alniacik U. (2022). The Moderating Role of Message Framing on the Links Between Organizational Identification and Unethical Pro-Organizational Behavior. *Management Research Review*.

[16] Yan H. M., Hu X. W., Wu C. H. (2021). When and How Can Organizational Punishment Stop Unethical Pro-Organizational Behaviors in Hospitality?. *International Journal of Hospitality Management*.

[17] Castille C. M., Buckner J. E., Thoroughgood C. N. (2018). Prosocial Citizens Without a Moral Compass? Examining the Relationship Between Machiavellianism and Unethical Pro-Organizational Behavior. *Journal of Business Ethics*.

[18] Hsieh H. H., Hsu H. H., Kao K. Y., Wang C. C. (2020). Ethical Leadership and Employee Unethical Pro-Organizational Behavior: A Moderated Mediation Model of Moral Disengagement and Coworker Ethical Behavior. *The Leadership & Organization Development Journal*.

[19] Nguyen C. M., Zhang L., Morand D. (2021). Unethical Pro-Organizational Behavior: A Moderated Mediational Model of Its Transmission From Managers to Employees. *Journal of Leadership & Organizational Studies*.

[20] Dou K., Chen Y., Lu J., Li J., Wang Y. (2019). Why and When Does Job Satisfaction Promote Unethical Pro-Organizational Behaviours? Testing a Moderated Mediation Model. *International Journal of Psychology*.

[21] Sheedy E., Garcia P., Jepsen D. (2021). The Role of Risk Climate and Ethical Self-Interest Climate in Predicting Unethical Pro-Organisational Behaviour. *Journal of Business Ethics*.

[22] Valle M., Kacmar K. M., Zivnuska S. (2019). Understanding the Effects of Political Environments on Unethical Behavior in Organizations. *Journal of Business Ethics*.

[23] Chen M., Chen C. (2021). The Moral Dark Side of Performance Pressure: How and When It Affects Unethical Pro-Organizational Behavior. *International Journal of Human Resource Management*.

[24] Ji X. Y. (2021). *Research on the Influence of Emotional Labor on Unethical Pro-Organizational Behavior of Medical Staff-The Mediating Effect of Organizational Identity*.

[25] Yan L. Y., Yuan J. Y., Qiang L. G., Rui L., Min Z. H. (2018). Research on Medical Staff Unethical Pro-Organizational Behavior From the Perspective of Goal Orientation in Performance Appraisal. *Journal of Xinxiang Medical University*.

[26] Ahmed M., Khan M. I. (2023). Beyond the Universal Perception: Unveiling the Paradoxical Impact of Ethical Leadership on Employees’ Unethical Pro-Organizational Behavior. *Heliyon*.

[27] Ajzen I., Kuhl J., Beckmann J. (1985). From Intentions to Actions: A Theory of Planned Behavior. *Action Control: From Cognition to Behavior*.

[28] Kan M. P. H., Fabrigar L. R., Zeigler-Hill V., Shackelford T. (2017). Theory of Planned Behavior. *Encyclopedia of Personality and Individual Differences*.

[29] Armitage C. J., Conner M. (2001). Efficacy of the Theory of Planned Behaviour: A Meta-Analytic Review. *British Journal of Social Psychology*.

[30] Zhou Y., Nie X., Lin Y., Pan S., Cao R., Zhang Y. (2025). Enhancing the Theory of Planned Behavior With Social Reactive Constructs in Explaining Adolescents’ Dietary Behaviors. *Appetite*.

[31] Tang L., Zhuang Z., Luo M., Cai Y., Lyu Q. (2025). Exploring the Determinants to Accept Dementia Screening Among Patients at High Risk of Dementia Based on the Theory of Planned Behavior: A Cross-Sectional Study. *Preventive Medicine*.

[32] Daher J., Mountjoy M., El Khoury D. (2025). An Online Nutrition Education Program Targeting Intentions and Related Determinants Towards Dietary Supplement Use: An Application of the Theory of Planned Behavior. *Nutrients*.

[33] Zhao Y. L. (2025). *Research on the Influencing Mechanism of Nurses’ Unsafe Behavior Intentions Based on TPB and 24Model*.

[34] Ning P., Chen X. Z., Zhan P. C., Wang L. J., Xiao Z. T., Li S. F. (2024). Qualitative Study on Behavioral Intention and Influencing Factors of PICU Nurses’ Management of Delirium. *Journal of Nursing Science*.

[35] Shi L. L., Liu D., Luo Y., Gao F. (2021). Influencing Factors of Ventilator Alarm Management of ICU Nurses: A Qualitative Research. *Chinese Nursing Research*.

[36] Sandelowski M. (2000). Whatever Happened to Qualitative Description?. *Research in Nursing & Health*.

[37] Tong A., Sainsbury P., Craig J. (2007). Consolidated Criteria for Reporting Qualitative Research (COREQ): A 32-Item Checklist for Interviews and Focus Groups. *International Journal for Quality in Health Care*.

[38] Hsieh H. F., Shannon S. E. (2005). Three Approaches to Qualitative Content Analysis. *Qualitative Health Research*.

[39] Lindgren B. M., Lundman B., Graneheim U. H. (2020). Abstraction and Interpretation During the Qualitative Content Analysis Process. *International Journal of Nursing Studies*.

[40] Graneheim U. H., Lundman B. (2004). Qualitative Content Analysis in Nursing Research: Concepts, Procedures and Measures to Achieve Trustworthiness. *Nurse Education Today*.

[41] World Medical Association (2013). World Medical Association Declaration of Helsinki: Ethical Principles for Medical Research Involving Human Subjects. *JAMA*.

[42] Steele L. M., Rees R., Berry C. M. (2024). The Role of Self-Interest in Unethical Pro-Organizational Behavior: A Nomological Network Meta-Analysis. *Journal of Applied Psychology*.

[43] Pohjanoksa J., Stolt M., Suhonen R., Löyttyniemi E., Leino-Kilpi H. (2019). Whistle-Blowing Process in Healthcare: From Suspicion to Action. *Nursing Ethics*.

[44] Crigger N., Godfrey N. (2014). Professional Wrongdoing: Reconciliation and Recovery. *Journal of Nursing Regulation*.

[45] Papinaho O., Häggman‐Laitila A., Pasanen M., Kangasniemi M. (2022). Disciplinary Processes for Nurses, From Organizational Supervision to Outcomes: A Document Analysis of a Regulatory Authority’s Decisions. *Journal of Nursing Management*.

[46] Liu X. L., Lu J. G., Zhang H., Cai Y. (2021). Helping the Organization but Hurting Yourself: How Employees’ Unethical Pro-Organizational Behavior Predicts Work-to-Life Conflict. *Organizational Behavior and Human Decision Processes*.

[47] Blair W., Kable A., Palazzi K., Courtney‐Pratt H., Doran E., Oldmeadow C. (2021). Nurses’ Perspectives of Recognising and Responding to Unsafe Practice by Their Peers: A National Cross-Sectional Survey. *Journal of Clinical Nursing*.

[48] Lian H., Huai M., Farh J. L., Huang J. C., Lee C., Chao M. M. (2020). Leader Unethical Pro-Organizational Behavior and Employee Unethical Conduct: Social Learning of Moral Disengagement as a Behavioral Principle. *Journal of Management*.

[49] Kong D. T. (2016). The Pathway to Unethical Pro-Organizational Behavior: Organizational Identification as a Joint Function of Work Passion and Trait Mindfulness. *Personality and Individual Differences*.

[50] Naseer S., Bouckenooghe D., Syed F., Khan A. K., Qazi S. (2020). The Malevolent Side of Organizational Identification: Unraveling the Impact of Psychological Entitlement and Manipulative Personality on Unethical Work Behaviors. *Journal of Business and Psychology*.

[51] Zhang Y., Zhu Y., Huang Z., Long L. (2024). Compensatory Effects of Loneliness: How and When Does Workplace Loneliness Promote Employees Unethical Pro-Organizational Behavior. *Current Psychology*.

[52] Wang X., Zheng X., Zhao S. (2022). Repaying the Debt: An Examination of the Relationship Between Perceived Organizational Support and Unethical Pro-Organizational Behavior by Low Performers. *Journal of Business Ethics*.

[53] Grabowski D., Chudzicka‐Czupała A., Chrupała‐Pniak M., Mello A., Paruzel‐Czachura M. (2019). Work Ethic and Organizational Commitment as Conditions of Unethical Pro‐Organizational Behavior: Do Engaged Workers Break the Ethical Rules?. *International Journal of Selection and Assessment*.

[54] Miao Q., Newman A., Yu J., Xu L. (2013). The Relationship Between Ethical Leadership and Unethical Pro-Organizational Behavior: Linear or Curvilinear Effects?. *Journal of Business Ethics*.

[55] Yang J. C., Lu L., Yao N., Liang C. C. (2020). Self-Sacrificial Leadership and Employees’ Unethical Pro-Organizational Behavior: Roles of Identification With Leaders and Collectivism. *Social Behavior and Personality: An International Journal*.

[56] Effelsberg D., Solga M., Gurt J. (2014). Transformational Leadership and Follower’s Unethical Behavior for the Benefit of the Company: A Two-Study Investigation. *Journal of Business Ethics*.

[57] Wiisak J., Suhonen R., Leino‐Kilpi H. (2023). Reasoning for Whistleblowing in Health Care. *Scandinavian Journal of Caring Sciences*.

[58] Blair W., Courtney-Pratt H., Doran E., Kable A. (2022). Nurses’ Recognition and Response to Unsafe Practice by Their Peers: A Qualitative Descriptive Analysis. *Nurse Education in Practice*.

[59] Suhonen R., Stolt M., Habermann M. (2018). Ethical Elements in Priority Setting in Nursing Care: A Scoping Review. *International Journal of Nursing Studies*.

